# Chemerin Normal Function and Roles in Metabolic and Nonmetabolic Disorders: An Up‐To‐Date Comprehensive Review

**DOI:** 10.1155/bri/1761697

**Published:** 2026-07-07

**Authors:** Noha A. Ahmed, Aida A. Hussein, Rehab G. Khalil, Nour Y. S. Yassin, Mohamed A. Abdelaziz, Ayman I. Geddawy, Osama M. Ahmed

**Affiliations:** ^1^ Physiology Division, Department of Zoology, Faculty of Science, Beni-Suef University, P.O. Box 62521, Beni-Suef, Egypt, bsu.edu.eg; ^2^ Physiology Division, Department of Zoology, Faculty of Science, Suez University, Suez, Egypt, suezuniv.edu.eg; ^3^ Immunology Division, Department of Zoology, Faculty of Science, Beni-Suef University, P.O. Box 62521, Beni-Suef, Egypt, bsu.edu.eg; ^4^ Department of Basic Medical Sciences, College of Medicine, Prince Sattam Bin Abdulaziz University, Al Kharj, 16273, Saudi Arabia, psau.edu.sa; ^5^ Department of Medical Physiology, Faculty of Medicine, Al-Azhar University, Cairo, Egypt, azhar.edu.eg; ^6^ Department of Pharmacology, Faculty of Medicine, Minia University, Minia, 61519, Egypt, minia.edu.eg

**Keywords:** chemerin, metabolic disorders, non-metabolic disorders, physiological functions, signaling pathways

## Abstract

Chemerin (retinoic acid receptor responder 2; RARRES2) is a secreted chemoattractant/adipokine that is expressed mainly in white adipose tissue, liver, and placenta and becomes bioactive after C‐terminal proteolytic processing into isoforms with distinct activities. This review summarizes (i) chemerin biosynthesis, processing, receptors, and signaling (CMKLR1/ChemR23, GPR1, and CCRL2), (ii) key physiological roles in immune cell trafficking, adipogenesis, glucose and lipid homeostasis, and vascular biology, and (iii) current evidence linking chemerin signaling to major metabolic disorders and selected nonmetabolic diseases. Overall, circulating chemerin is frequently elevated in obesity, insulin resistance, metabolic syndrome, Type 2 diabetes, nonalcoholic fatty liver disease, and hypertension and often correlates with inflammatory markers; however, heterogeneity in study design, confounding, and limited isoform‐specific measurements complicate causal inference and diagnostic validation. Beyond metabolism, chemerin signaling has been implicated in inflammatory conditions (e.g., rheumatoid arthritis and psoriasis) and multiple cancers with tumor type–specific pro‐ or antitumor effects. Standardized assays, isoform‐resolved measurements, and prospective studies are needed to clarify disease mechanisms and determine the clinical utility of chemerin as a biomarker or target.

## 1. Introduction

Chemerin was first discovered by Nagpal et al. in 1997. Over a thousand publications about chemerin have been written in the last 3 decades following its first discovery. Chemerin is released in an inactive precursor, prochemerin [1]. To become an effective chemoattractant, prochemerin must undergo proteolytic processing of its C‐terminus [2].

Chemerin has a significant impact on lipid metabolism and adipogenesis. In addition, it influences insulin signaling, steroidogenesis, thermogenesis, and inflammation. As such, it probably has a role in a number of metabolic and cardiovascular conditions, such as diabetes, atherosclerosis, and hypertension [3, 4].

Chemerin is not only implicated in the pathophysiology of metabolic disorders but also involved in many nonmetabolic disorders [2, 5–7].

In this review, we first outline chemerin synthesis, proteolytic activation, receptor biology, and downstream signaling. We then summarize well‐defined physiological roles and mechanisms of chemerin before discussing disease associations in dedicated sections focused on metabolic disorders (obesity, diabetes, metabolic syndrome (MetS), hypertension, and cardiometabolic disease) and selected nonmetabolic conditions with substantial emerging evidence (inflammatory skin/joint disorders and cancer).

The search strategy combined controlled vocabulary terms and free‐text keywords using Boolean operators (AND/OR). The primary search terms included the following: “chemerin,” “retinoic acid receptor responder 2 (RARRES2),” “adipokine,” “metabolic disorders,” “obesity,” “type 2 diabetes,” “insulin resistance,” “inflammation,” “psoriasis,” “lupus erythematosus,” “cancer,” “angiogenesis,” “vascular endothelial growth factor (VEGF),” “matrix metalloproteinases (MMPs),” “interleukin‐6 (IL‐6),” “p38 mitogen‐activated protein kinase (p38 MAPK),” and “extracellular Signal‐Regulated Kinase 1 and 2 (ERK1/2) signaling.” We searched multiple databases, including PubMed, Scopus, and Web of Science, to gather a wide range of peer‐reviewed articles on the topic. Only studies published in English were included to maintain consistency and accessibility of content. Additionally, reference lists of selected articles were reviewed to identify any relevant studies not captured in the initial search.

The selection strategy focused on studies addressing the biochemical functions of chemerin and its involvement in metabolic and nonmetabolic disorders. Given the broad scope, our goal was to provide a balanced overview to cover critical aspects of molecular mechanisms underlying chemerin’s physiological functions and pathological roles. The review integrates findings related to chemerin’s involvement in insulin resistance, adipose tissue dysfunction, inflammatory skin diseases, and cancer progression, highlighting its context‐dependent proinflammatory and anti‐inflammatory activities. The potential of chemerin as a biomarker and therapeutic target in metabolic and nonmetabolic disorders is critically discussed.

## 2. Chemerin Synthesis and Release

Chemerin, alternatively referred to as tazarotene‐induced gene 2 (TIG2) or RARRES2, is structurally associated with the cathelicidin/cystatin protein family and was originally described by the synthetic retinoid tazarotene as an overexpressed gene in psoriatic skin [1, 8]. Many processes, such as expression, secretion, processing, and signaling events, tightly control chemerin signaling. These regulatory processes must be precisely coordinated in order to determine chemerin levels, localization, and, eventually, behavior [9, 10].

Chemerin is most abundant in the placenta, liver, and white adipose tissue (WAT) and, to a lesser degree, in many other tissues, including the lung, brown adipose tissue, heart, ovary, kidney, skeletal muscle, and pancreas [11]. Chemerin expression in adipocytes is abundant in WAT relative to the stromal vascular fraction [12]. The main source of circulating chemerin is thought to be WAT and in addition to the liver. The serum levels of chemerin in mice fluctuate in a diurnal manner with peak and trough cycles, much like other adipokines as leptin and adiponectin [13]; however, these oscillations may be small in humans [14].

In healthy, lean populations, serum and plasma concentrations of total circulating chemerin range from 90 to 200 ng mL^−1^ [10, 15]. Generally speaking, chemerin levels are higher in women and older adults than in men and younger ones [15, 16], although these patterns have not been recorded in all studies [16, 17]. Initially, before the inactive 18‐kDa precursor, prochemerin, is released, the 163 amino acid protein preprochemerin, which has an N‐terminal signal sequence (20 aa), is divided to create chemerin (Chem‐163) [8]. It is thought that the majority of chemerin in circulation is in the comparatively inactive form of prochemerin, and in order to produce local biological effects, bioactive chemerin is processed by proteolysis [8, 9].

Prochemerin cleavage via extracellular proteolysis is necessary for the production of chemerin. Additionally, it was demonstrated that chemerin needed to be separated from prochemerin to become active [18]. Prochemerin C‐terminal proteolysis processing by various proteases creates at least six isoforms, involving chemerin K158 (low activity), chemerin S157 (highest activity), chemerin F156 (high activity), chemerin A155 (no activity), chemerin F154 (no activity), and chemerin G152 (no activity) [19]. Several proteases have been implicated in activating this protein, including plasmin, elastase, and inflammatory serine protease tryptase. Various proteases do not cleave the peptide at the exact location, which is interesting [20, 21].

Furthermore, one of the most unknown aspects of chemerin in the field of epidemiology is isoforms, which are produced by variations in cleavage points. Chemerin A155 is the identified serum chemical reported by Zhao et al. [8]. The most potent version of chemerin S157 in the serum, however, has values for 50% effective concentration (EC50) = 1.17 nM for calcium mobilization and EC50 = 3.15 nM for percent migration, which cause chemotaxis and calcium mobilization in the murine pre‐B lymphoma cell line L1.2 [8]. Chemerin K158 is also prevalent in cerebrospinal fluid (CSF) and synovial fluid [8, 22]. But most interestingly, in 2021, Su et al. [23] showed that the creation of chemerin‐15 (mouse, A140‐A154) and its distinct proteolytic cleavage showed anti‐inflammatory qualities on the receptor [23]. The medical community has not yet identified these isotypes in their various pathologies, which is extremely limited to the development of this study but also presents an opportunity to explain many epidemiological issues. The wide variation in the distribution and efficacy of isoforms is already astounding [8, 24].

The proteases, carboxypeptidase B (CPB), carboxypeptidase N (CPN), and mast cell chymase, are of special relevance to chemerin. CPB and CPN have the ability to change the active chemerin‐9 into the inert chemerin K158. These proteases can be coupled with plasmin, which generates the inert chemerin K158, to create an active product [25]. It is also noteworthy that mast cell chymase can change the active chemerin S157 into the inert form of chemerin F154 [26, 27].

Chemerin is a newly identified adipokine that has a role in energy metabolism, inflammatory control, and adipogenesis [28, 29]. Adipokine chemerin controls the function of innate immune cells in obesity and Type 2 diabetes (T2D) and may be associated with inflammation. Therefore, a possible connection between chemerin and inflammatory proteins was evaluated. Patients with liver cirrhosis had their portal venous, hepatic venous, and systemic venous blood tested for chemerin because visceral fat causes systemic inflammation [8, 17].

**Figure 1:**
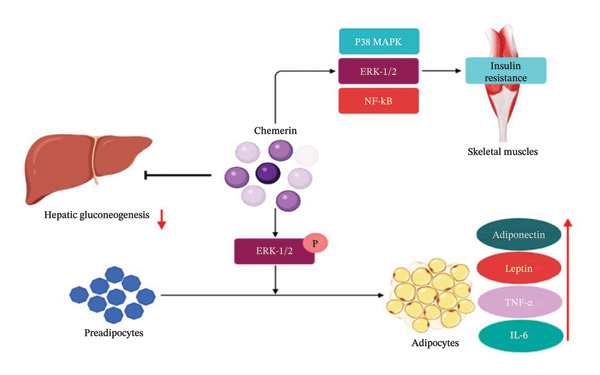
Effects of chemerin on insulin resistance and various adipocytokines, such as leptin, adiponectin, TNF‐α, and IL‐6, involved in controlling metabolic and inflammatory responses, through ERK1/2 phosphorylation.

Chemerin is a newly identified adipokine implicated in inflammation, adipogenesis, and energy metabolism [30–32]. Chemerin was proposed as a likely connection between the advancement of T2D and obesity [10, 30], and both T2D and obese individuals with leptin resistance showed elevated gene expression and serum levels [15]. On the other hand, chemerin gene expression in rat WAT reduced after food restriction [33], and the administration of chemerin has been associated with modulatory effects on a number of adipokines that regulate inflammation and metabolism, including interleukin‐6, adiponectin, and leptin [8, 29, 31] (Figure 1).

It has recently been discovered that adipose tissue has high expression of chemerin [9, 34]. Chemerin is an orphan G protein–coupled receptor (GPR) agonist that is expressed by innate immune system cells and chemokine‐like receptor 1 (CMKLR1) [29, 35, 36]. As such, it can further connect inflammation and obesity [16, 37]. Chemerin is released as an inactive precursor and is cleaved by serine coagulation proteases, fibrinolytic cascades, and inflammatory cascades. Chemerin causes these cells to chemotactic, indicating that it and its receptor play a part in the innate immune response [18, 23].

Obesity enhances the expression of the adipokine chemerin in adipose tissue and its systemic levels. Despite the significant abundance of chemerin in adipocytes, the molecular processes underlying its further elevation in obesity have not been explained. The production of dysregulated adipokines is facilitated by adipocyte hypertrophy, and we postulated that excessive loading triggers the production of chemerin using free fatty acids (FFAs). The differentiation of 3T3‐L1 cells in the presence of FFA further increased the amount of chemerin, which was expressed in mature adipocytes. TNF and IL‐6 were produced by FFA, but the amounts were insufficient for chemerin to increase them. Sterol regulatory element–binding protein 2 (SREBP2) was activated in those cells, indicating a possible cholesterol deficiency. Supplementing with mevalonate reversed the effect of lovastatin’s inhibition of cholesterol production, which enhanced chemerin and activated SREBP2. Chemerin‐induced and basal FFAs were reduced by SREBP2 knockdown. The electrophoretic mobility shift assay (EMSA) verified that the chemerin promoter at the SREBP location was bound by 3T3‐L1 nuclear adipocyte proteins [38]. Higher systemic levels appear to be obtained from adipocytes, as SREBP2 was activated and chemerin was produced in the adipose tissue of animals given a high‐fat diet. Both processes are equally important, as evidenced by the equivalent effectiveness of lipopolysaccharide‐mediated chemerin elevation and FFA induction. Bigger fat expression in mice given a high‐fat diet may indicate a bigger number of obesity‐resident adipose tissue macrophages, and the aforementioned incubations have not changed CMKLR1. According to recent studies, adipocyte hypertrophy, chemerin production, and chronic inflammation are all equally important [10, 38].

Recently identified as an adipokine, chemerin has a positive correlation with markers of MetS, such as systemic triglycerides and the index of body mass. Additionally, chemerin has a positive correlation with the levels of inflammatory cytokines in the blood [17, 23, 39]. Chemerin in supernatants is inversely correlated with the insulin sensitivity of these cells, and obese donor adipose tissue explants produce higher quantities of chemerin than lean control explants [40, 41]. Obesity is characterized by persistent low‐grade inflammation and elevated levels of proinflammatory cytokines in the body and adipose tissue. TNF raises the amount of chemerin in the bloodstream in mice, and proinflammatory cytokines like IL‐1 and TNF cause adipocyte chemerin to be synthesized [42–44]. Therefore, in human and mouse models of obesity, elevated systemic chemerin was proposed to originate from inflammatory cytokines, further promoting its synthesis in adipocytes [10, 23, 43] (Figure 1).

Chemerin is an agonist of the G protein–coupled CMKLR1 that is expressed by adipocytes, allowing for its autocrine and/or paracrine actions [15, 31, 45]. In addition to CMKLR1 (as known as chemerin receptor 1 or ChemR23), the secreted chemerin activates two other receptors: GPR1 (chemerin receptor 2 [ChemR2]) and chemokine CC‐motif receptor‐like 2 (CCRL2, chemerin receptor 3 [ChemR3]) [19].

Recombinant chemerin precludes the in vitro uptake of insulin‐induced glucose in adipocytes [42, 46], whereas during a glucose tolerance test, the application of recombinant chemerin has no effect on the incorporation of adipose tissue glucose in mice [30, 47]. The expression of CMKLR1 was first identified in immune cells and thereafter in skeletal [48] muscle cells. Chemerin promotes the chemotaxis of Tan et al. [3] macrophages and dendritic cells, although insulin resistance is enhanced in the latter [49, 50]. However, it has not been proved to date whether CMKLR1 mediates chemerin‐induced insulin resistance in these cells. Importantly, chemerin contains neither macrophages nor skeletal muscle cells [40, 51].

Chemerin promotes leukocyte migration to inflammation sites and also increases inflammatory signaling in chondrocytes, indicating chemerin’s role in joint inflammation [6, 52]. Chemerin, often referred to as TIG2 protein, is a chemotactic protein that has been linked to the attraction of immune cells, such as dendritic cells and macrophages [53, 54]. The chemotactic action of chemerin is mediated by its interaction to the CMKLR1 [53, 55]. According to more recent studies, articular chondrocytes, at least in murine chondrocytes, express CMKLR1 and even produce interleukin‐1 beta (IL‐1β)‐induced chemerin [23, 56, 57]. Chemerin stimulates the synthesis of TNF, IL‐6, IL‐1β, MMP‐1, and MMP‐8 in human articular chondrocytes. This chemokine also increases the synthesis of interleukin‐8 (IL‐8), MMP‐2, MMP‐3, and MMP‐13 at higher concentrations. These findings suggest a connection between chemerin and cartilage deterioration and joint inflammation [56, 58].

Chemerin tends to be primarily produced by adipocytes, with an increase in obesity in its production and serum levels [23]. In adipocytes, proinflammatory cytokines, and lipopolysaccharides stimulate the production of chemerin, and in mice, TNF raises the amount of chemerin in the blood [30, 59].

Because systemic chemerin is higher in obese and T2D patients, it can affect insulin signaling in adipocytes and skeletal muscle cells, which can result in insulin resistance [5, 30, 38]. Chemerin correlates positively with circulating inflammatory cytokine levels in obesity, demonstrating that elevated levels of chemerin are associated with inflammation [23, 30]. Notably, rheumatoid arthritis (RA) patients have also been found to have higher amounts of chemerin in inflammatory bodily fluids and tissues, including synovial joint fluid, psoriatic skin, and ascites [18, 60]. The fibroblasts of murine 3T3‐L1 express CMKLR1, while chemerin is not present in either cell lysate or supernatants [38, 61]. This indicates that chemerin is capable of responding to fibroblasts [52, 61]. Prior research revealed that human synovial fibroblasts (SFs) express both chemerin and its receptor. Because chemerin increases Toll‐like receptor (TLR)‐4 expression and causes CC‐chemokine ligand (CCL)‐2 to be released in SFs, it is hypothesized that this protein plays a part in innate immune system‐related joint inflammation [6, 52].

Chemerin is a chemical attractant for immature human dendritic cells (DCs), macrophages, and natural killer cells (NK cells) and a ligand for ChemR23 (also known CMKLR1) [62, 63]. The ChemR23 ligand is a novel extracellular mediator that is expressed by NK cells, macrophages, and immature mDCs and pDCs. It is a GPR [18, 37, 64]. High levels of active chemerin were discovered in a variety of inflammatory disorders, including human RA, inflammatory ascites, and inflammatory illnesses that recruit cells expressing ChemR23 [23, 37, 65]. Chemerin was characterized at subnanomolar concentrations as a strong chemoattractant factor [65, 66]. Proteolytic removal of the final six or seven amino acids transforms the protein into a complete ChemR23 agonist. The protein is released as an inactive precursor called prochemerin [67, 68].

It has been demonstrated that chemerin is produced from prochemerin by extracellular proteases, including neutrophil‐derived cathepsin G and elastase, suggesting that the treatment acts at areas of inflammation [29, 69]. Increased synthesis of chemerin was observed in psoriasis skin [70] and lupus erythematosus skin lesions [65, 71, 72], in addition to the high expression of ChemR23 on pDCs, which are thought to be essential for skin inflammatory processes [23, 60, 73]. For this reason, the new chemerin/ChemR23 combination offers a promising option to control complex DC movement in inflammatory settings. Furthermore, we showed that chemerin can exhibit strong anti‐inflammatory qualities based on ChemR23 in vivo in addition to being an efficient chemoattractant to antipresenting cells (APCs) in a mouse model of acute lung damage [6, 62].

Serum levels of chemerin are higher in obese patients and positively correlate with several elements of MetS, according to a thorough review of human experimental data [3].

Following proteolytic cleavage of a signal peptide as a 143 amino acid (18 kDa), chemerin is translated as a preprotein 163 amino acid that is released proprotein [9, 53]. Plasmin, CPs, or serine proteases are needed to process coagulation proteases and fibrinolytic and inflammatory cascades at the extracellular C‐terminus of this proprotein since it has limited biological activity [74]. Interestingly, the source of chemerin affects the degree of C‐terminal cleavage. Six, eight, and nine C‐terminal amino acids are absent from chemerin from human fluid, serum, and hemofiltrate ovarian ascites, respectively [8, 19, 20]. Proteolytic processing of chemerin is an essential regulatory mechanism that may help determine the local and systemic amounts of bioactive chemerin. One important topic of research is the significance of chemerin control from a physiological and pathological standpoint. Curiously, many of the known biological actions of chemerin can be substantially recapitulated by short peptides that are synthetic variants or identical to the 9‐15 C‐terminal amino acids of chemerin 20‐1571 [75]. This suggests that the rest of the protein is dispensable and functionally inert, albeit an implausible assumption. It will take more research to ascertain whether the N‐terminal region plays a role in the interaction between chemerin and GPR1 and CCRL2 or in the creation of multimeric chemerin complexes [30, 76].

## 3. Chemerin Receptors and Signaling Pathways

Three recognized receptors are by chemerin include CMKLR1, chemerin receptor 1 or ChemR23, chemokine (CC‐motif, ChemR2) receptor‐like 2 (CCRL2), and GPR1, ChemR3 [19]. Among them, the chemerin receptor 1, CMKLR1 or ChemR23, is the most important chemerin receptor. Adipose tissues, muscle tissues, endocrine tissues, the female reproductive system (placenta, endometrium, etc.), lung, and a variety of innate immune cell types (plasmacytoid dendritic cells, macrophages, etc.) all exhibit high expression of CMKLR1. In the brain (choroid plexus), esophagus, skin, placenta, adrenal gland, testis, ovary, gallbladder, and adipose tissues, GPR1 is strongly expressed. Although it is broadly distributed, the lung, gastrointestinal tract, adipose tissues, breast, placenta, and immune cells (macrophages, etc.) have the highest quantities of CCRL2 mRNA [77].

In response to chemerin, the receptor CMKLR1 activates intracellular signaling molecules and participates in metabolic and inflammatory responses by causing intracellular Ca^2+^ release, phosphorylation of MAPKs, such as p42/p44 and p38 MAPK, and inhibition of cyclic adenosine monophosphate (cAMP) accumulation through binding to G protein–coupled heterotrimers. These signaling events lead to diverse cellular responses, including chemotaxis, adipogenesis, and the modulation of inflammatory processes [31, 78].

On the other hand, CCRL2 acts as a nonsignaling receptor that binds chemerin with no initiating signal transduction. Presenting chemerin to cells that express CMKLR1 is the main function of this receptor, which raises local chemerin concentrations and promotes CMKLR1‐mediated reactions. Since leukocytes, endothelial cells, and other immune cells express CCRL2, its function is especially important in inflammatory situations [79, 80].

**Figure 2:**
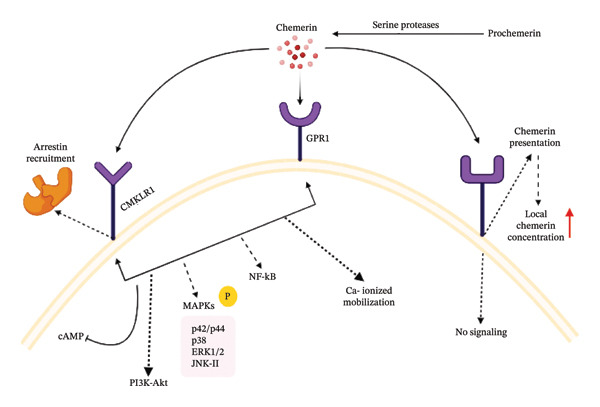
Pathways for the effects of chemerin on apoptosis, migration, invasion, and metastasis of tumors. The signaling pathways activated by chemerin through its receptors CMKLR1, GPR1, and CCRL2. Key components include CMKLR1: chemokine‐like receptor 1, GPR1: G protein–coupled receptor 1, CCRL2: CC‐chemokine receptor‐like 2, MAPK: mitogen‐activated protein kinase, cAMP: cyclic adenosine monophosphate, ERK: extracellular signal‐regulated kinase, JNK: c‐Jun N‐terminal kinase, NF‐κB: nuclear factor kappa‐light‐chain‐enhancer of activated B cells, and PI3K‐Akt: phosphoinositide 3‐kinase‐Akt.

Despite having structural similarities to CMKLR1, GPR1, the third type of chemerin receptor, has unique functional characteristics. Despite having a high affinity for chemerin, GPR1 only weakly mobilizes Ca2+ in comparison with CMKLR1. Arrestin recruitment, as opposed to conventional G protein–mediated pathways, is the main signaling mechanism of GPR1. Long‐term cellular processes are regulated by GPR1 binding to chemerin, which results in more persistent cellular responses such as receptor internalization and the activation of alternate pathways like MAPKs [78, 81]. Since GPR1 is mostly expressed in the central nervous system and some peripheral organs, it may play a part in regulating metabolic and neuroendocrine processes. While the precise physiological and pathological activities of GPR1 remain to be determined, its unique expression pattern suggests functions distinct from those mediated by CCRL2 and CMKLR1 [78, 82] (Figure 2).

## 4. Physiological Functions and Mechanisms of Chemerin

Chemerin is a multifunctional adipokine/chemokine whose actions depend on tissue source, proteolytic processing (isoform profile), and receptor context (CMKLR1, GPR1, and CCRL2). Physiological roles described below provide the mechanistic basis for the disease associations discussed in Sections 5 and 6.

### 4.1. Immune Surveillance and Regulation of Inflammation

Chemerin was originally characterized as a chemoattractant that directs the migration of innate immune cells, including dendritic cells, macrophages, and NK cells, toward sites of tissue injury and infection [9, 30, 83]. After secretion as prochemerin, local proteases generate active C‐terminal isoforms that bind primarily to CMKLR1, triggering cell migration and context‐dependent inflammatory signaling [2, 74]. Depending on the experimental model and isoform balance, chemerin has been reported to exert both proinflammatory and anti‐inflammatory effects, emphasizing the importance of the microenvironment and receptor expression profile [84].

### 4.2. Adipose Tissue Biology, Lipid Metabolism, and Insulin Signaling

In adipose tissue, chemerin is expressed by adipocytes and stromal cells and can act in autocrine/paracrine and endocrine manners. Experimental studies indicate that chemerin participates in adipocyte differentiation, lipid handling, and crosstalk with other adipocytokines, and elevated chemerin can impair insulin‐stimulated glucose uptake in adipocytes [42, 46]. Importantly, chemerin bioactivity differs between tissues and circulation because isoform distributions are shaped by local protease activity, which may explain discrepancies across studies relying on total chemerin measurements [8].

### 4.3. Vascular Biology and Angiogenesis

Chemerin signaling has been implicated in endothelial and vascular smooth muscle function. In experimental systems, chemerin can modulate angiogenesis and vascular remodeling and may influence vascular tone through CMKLR1‐dependent pathways [85, 86]. These physiological activities may contribute to the observed links between chemerin, blood pressure regulation, and cardiometabolic risk discussed in Section 5.

### 4.4. Skin, Bone, and Other Physiological Contexts

Chemerin is expressed in several nonadipose tissues and may contribute to tissue homeostasis beyond metabolism. For example, chemerin‐derived peptides show antimicrobial activity in the epidermis and may participate in barrier defense [87]. Chemerin has also been linked to bone‐cell differentiation pathways, suggesting a role in the balance between osteogenesis and adipogenesis within the bone marrow niche [88].

Collectively, these physiological functions highlight chemerin as a mediator at the intersection of metabolism, immunity, and vascular biology. In the following sections, we summarize clinical and experimental evidence supporting chemerin involvement in metabolic and selected nonmetabolic disorders.

## 5. Association of Chemerin With Metabolic Diseases

The association of chemerin with different metabolic conditions is shown in Table 1. The other associated functional and metabolic indices were also reported.

**TABLE 1:** Summary of representative human evidence linking circulating chemerin to major metabolic conditions (selected examples).

Condition	Typical finding	Commonly reported associations	Notes/limitations
Obesity	↑ total circulating chemerin	BMI/adiposity, triglycerides, CRP, and impaired insulin sensitivity [16, 89, 90]	Assay and isoform heterogeneity; adipose vs liver contribution
Type 2 diabetes	Often ↑	Homeostasis model assessment of insulin resistance (HOMA‐IR), glycated hemoglobin (HbA1c), cardiometabolic risk markers [45, 91]	Confounding by obesity and inflammation; causality uncertain
Metabolic syndrome	Often ↑	Clustered cardiometabolic risk factors [92–94]	Definition variability across studies
NAFLD	Often ↑	HOMA‐IR and steatosis severity [95–101]	Liver produces chemerin; need tissue/isoform‐resolved data [15, 51]
Hypertension/vascular dysfunction	Often ↑	Blood pressure and vascular tone markers [102, 103]	Medication and renal function confounding; mixed results
Atherosclerosis/cardiometabolic disease	Context‐dependent	ChemR23 detected in lesions; nitric oxide (NO)‐cyclic guanosine monophosphate (cGMP) signaling changes [103, 104]	Total chemerin may not reflect bioactive isoforms

### 5.1. Chemerin and Obesity

Overweight and obesity pose serious health risks to the world. The body mass index (BMI) has a positive correlation with cardiovascular mortality, and being overweight is the known cardiovascular risk factor [10]. Particularly in the visceral compartment, WAT is now regarded as an active endocrine organ that releases a variety of physiologically active chemicals called adipokines, rather than just being a tissue that stores energy. Insulin sensitivity, inflammation, and glucose/lipid metabolism are all thought to be significantly influenced by adipokines. According to the intricate interactions between adipokines, obesity is frequently accompanied by chronic low‐grade inflammation and persistently heightened oxidative stress [10, 105]. Insulin resistance is also triggered by the imbalance between oxidative stress and antioxidant defense and results in increased atherosclerosis [16, 106].

Additionally, oxidative stress is a symptom of dyslipidemia linked to obesity, which causes cardiovascular illnesses by decreasing left ventricular function and aortic flow, which in turn increases the degree of myocardial necrosis in experimental settings [107, 108]. Chemerin is a new biologically active adipokine that has not yet been fully understood. It is also known as RARRES2 or TIG2. The protein is highly expressed in adipose tissue, as is its receptor (CMKLR1 or ChemR23) [108]; furthermore, it has been revealed that chemerin controls adipocyte metabolism and differentiation in an autocrine/paracrine way [16]. For immune cells including dendritic cells, NK cells, and macrophages, chemerin also serves as a chemoattractant [6]. Its precise impact on inflammation is yet unknown because it may also act as a pro‐ and anti‐inflammatory protein [23, 32].

Recent evidence shows that chemerin may contribute to the development of obesity and MetS [16, 89]. Chemerin can also impede the uptake of glucose and promote insulin resistance [67], and its level has been documented to be positively correlated with BMI and markers of human inflammation and MetS. Genetic studies also showed that chemerin may be involved with increased lipogenic activity in adipose tissue homeostasis, reporting that aged ChemR23 knockout mice were vulnerable to developing mild obesity without significant adipocyte differentiation defects [10]. Additionally, the relationship between chemerin and traditional adipokines like leptin and adiponectin has not yet been clarified. Proinflammatory reactions have been linked to leptin, and it has been demonstrated that body fat raises its serum levels [59]. In fact, leptin has been shown to contribute to the development of heart failure in rats with experimental myocardial infarction, likely by increasing the proinflammatory cytokine intramyocardial expression [109, 110]. Adiponectin levels, by comparison, decrease in obesity and are inversely linked with the risk of myocardial infarction [111]. In addition, adiponectin also enhances the sensitivity of insulin and the development of anti‐inflammatory cytokines [112].

It has been shown that a large proportion of obese individuals with normal insulin sensitivity are considered nondiabetic. In a recent report, metabolically healthy obese people, relative to metabolically unhealthy insulin‐resistant counterparts, have a lower risk of mortality and cardiovascular disease [113, 114]; however, obese individuals still have a greater risk of T2D [115]. This suggests that factors such as oxidative stress, antioxidant status, chronic inflammation, and adipokine interaction may have an impact on the clinical outcome in obese people in addition to basic calorie imbalance. Overweight and obesity are risk factors for a number of diseases, including cancer and T2D.

WAT is a significant source of adipokines, a complex collection of proteins with various roles. Among these proteins, chemerin has a higher systemic level of obesity. As a key player in metabolic health, chemerin controls adipogenesis, insulin sensitivity, and the immune system, among other physiologic and pathological processes. Most serum chemerin has no biological activity. Different proteases are involved in the C‐terminal processing of chemerin, which results in a variety of isoforms with varying activity levels. The distribution of chemerin variants was examined in the plasma and adipose tissues of mice and lean and obese people. Utilizing the Tango bioassay, suitable for tracking beta‐arrestin 2 pathway activation, the ex vivo activation of chemerin receptors by systemic chemerin was assessed. Additionally, the expression of chemerin receptors in skeletal, hepatic, and adipose tissue was evaluated [84, 116].

The protein chemerin plays a role in adaptive and innate immunity and is a chemoattractant for immune cells [9, 30, 83]. It controls angiogenesis, adipogenesis, and energy metabolism, demonstrating this protein’s multifaceted role [29, 117]. Positive associations between systemic chemerin and phenotypes associated with obesity, such as insulin resistance, BMI, and serum triglycerides, indicate that this adipokine has a role in metabolic diseases. The ex vivo activation of chemerin receptors by systemic chemerin was evaluated using the Tango bioassay, which is appropriate for monitoring beta‐arrestin 2 pathway activation. The expression of chemerin receptors in adipose, hepatic, and skeletal tissue was also evaluated [29].

Interestingly, less detectable adipose tissue macrophages were detected. While this implies enhanced insulin sensitivity, Akt phosphorylation caused by insulin has been decreased in the fat tissue [118]. A separate study shows that recombinant chemerin injection decreased serum insulin and tissue glucose uptake in obese mice but had no effect on animals of normal weight. Chemerin overexpression was found to induce muscle insulin resistance in low‐density lipoprotein (LDL) receptor–deficient mice, but not in liver or gonadal fat. There was no improvement in body weight, serum lipid levels, and atherosclerosis severity [119, 120].

Because adipokine chemerin is released by adipocytes, hepatocytes also generate substantial protein levels [90, 121]. Serum chemerin is increased in overweight/obesity, and in some but not all of the patient cohorts examined, correlations with obesity‐related characteristics, such as low‐grade inflammation, blood pressure, and insulin resistance, were established [122]. Therefore, the associations between systemic levels of chemerin and the MetS are not completely resolved [122]. Serum chemerin levels are inheritable with genetic factors being linked to around 16%–25% of variations. RARRES2 gene polymorphisms were linked to increased levels of systemic chemerin, visceral fat mass, and a higher risk of MetS [123].

In chronic inflammatory diseases, positive associations were identified between systemic chemerin and inflammatory cytokines and CRP [23]. Psoriasis, inflammatory bowel disease, RA, and chronic kidney illness all have pathophysiologies that heavily rely on chemerin [8]. More recent studies show a role of chemerin in cancer, and both pro‐ and anticarcinogenic effects have been reported [124, 125]. Chemerin was shown to suppress the growth of hepatocellular carcinoma but to increase the migration of squamous cell carcinoma [126, 127].

Chemerin isoform distribution in adipose tissue differed entirely from that of the circulating versions. While adipose tissues had more processed chemerin, prochemerin was released into the bloodstream. The distribution of isoforms was unaffected by obesity in either mice or humans. The life period of increased adipogenesis may be the reason why age had the greatest impact on the quantity of chemerin isoforms in mouse fat tissue [10]. Chemerin’s function in physiological and pathological processes has been demonstrated in a number of clinical and experimental investigations. However, the exact role of chemerin is still unknown. Despite an increase in systemic chemerin, its bioactivity remains unchanged in fat mice and humans. Very little is known about the physiologic role of GPR1 or its signal transduction pathways. A detailed description of the physiologic action of the chemerin isoforms binding to this receptor has not yet been provided. Obesity appears to change the way chemerin is processed. We have yet to identify the specific proteases implicated in this process. The C‐terminal amino acids of this adipokine are mimicked by synthetic peptides made from chemerin, which reduce inflammation and improve phagocytosis [19, 29], and can be used as therapeutic agents to treat metabolic diseases and probably more chronic inflammatory disorders [45].

### 5.2. Chemerin and Diabetes Mellitus

#### 5.2.1. T2D Mellitus and Insulin Resistance

Human insulin and T2D have been linked to novel adipokines, including dipeptidyl peptidase 4 (DPP4), vaspin, omentin, retinal binding protein‐4 (RBP‐4), fibroblast growth factor 21 (FGF21), and adipocyte fatty acid binding protein (A‐FABP) [128]. Additionally, a number of clinical research studies have examined the relationship between chemerin and diabetes levels. Notwithstanding the fact that systemic chemerin was considerably higher in T2D patients than in normal‐weight controls in the Caucasian population, without other metabolic problems [16], in Asian T2D patients, it remained unchanged or even declined [129]. Notably, an observational study shows that systemic chemerin elevation precedes the onset of T2D [16], indicating that chemerin could serve as an early diagnostic biomarker for T2D.

Additionally, linear regression studies demonstrate a cross‐sectional relationship between systemic chemerin in T2D patients and blood pressure, triglycerides, homeostasis model assessment of insulin resistance (HOMA‐IR), HbA1C, BMI, waist–hip ratio, age, and 2‐h plasma glucose in patients of all races [130, 131]. In obese individuals with T2D, the elevation of systemic and local chemerin in adipose tissue is greatly aggravated. However, the connection between circulating chemerin and gestational diabetes mellitus (GDM) has up until now been highly contentious [16, 132]. The upregulation of both systemic and local chemerin is common in people with T2D, especially those with MetS.

Wittamer et al. [18] identified chemerin as an adipokine and described its activity in modulating adipogenesis and adipocyte metabolism, which is correlated with MetS [18]. Not all studies have found an association between chemerin and GDM circulating. Yang et al. investigated that circulating chemerin was significantly elevated in women with GDM compared to controls. At the same time, Sadia et al. did not find a substantial correlation between the concentration of chemerin and GDM [16, 132].

#### 5.2.2. GDM

Different levels of the first recognized glucose intolerance during pregnancy are the hallmark of GDM, which affects 4%–18% of pregnant women based on various diagnostic criteria and ethnic origin [133]. The pathophysiologic process of GDM is comparable to that of T2D, involving insulin resistance, oxidative stress, and systemic inflammation [134]. Pregnant women with pre‐existing β‐cell abnormalities may also be unable to adjust to the increased insulin demand during pregnancy if they develop GDM [135, 136], and β‐cell malfunction and consequent insulin resistance are thought to be linked to systemic inflammation in diabetes individuals [137]. It is also clear, regardless of perspective, that chronic low‐grade inflammation and insulin resistance are important factors in the development of GDM [16].

A novel cytokine released mainly by WAT, chemerin was once thought to be a chemotactic factor generated during inflammation. However, more recent research has shown that it is actually an adipokine that controls adipose metabolism and energy balance [88]. Compared to healthy controls, serum levels of chemerin were shown to be considerably higher in individuals with biopsy‐proven NAFLD [138]. Furthermore, increased hepatic chemerin mRNA expression in human NAFLD was found to be independently linked to hepatocyte ballooning, inflammation, steatosis, and liver fibrosis [139]. Even after controlling for waist circumference, a recent population‐based investigation found a correlation between elevated chemerin levels, MetS, and inflammation [16, 140].

More significantly, chemerin is an independent indicator T2D and the risk of cardiovascular events [45, 91]. Recent studies have also indicated that chemerin may play a significant role in GDM’s pathogenetic mechanism. Studies of the relationship between circulating levels of chemerin and GDM have, however, yielded contradictory results [75, 141].

#### 5.2.3. T1D Mellitus and Microvascular Complications

T1D mellitus is one of the most prevalent chronic disorders in children that may result from micro‐ or macrovascular problems. Diabetic renal disease, often known as nephropathy, is a common consequence of diabetes mellitus that is clinically silent and the sole anomaly that may be detected because of microalbuminuria [142]. Chemerin contributes to the metabolism of glucose and lipids. Insulin resistance and systemic inflammation have been associated with elevated levels of this peptide [23]. It is interesting to note that elevated blood chemerin levels have been observed in T1D patients. These elevated levels can be regarded as promising adipokines in the development of diabetic problems; hence, testing serum chemerin in children with diabetes is helpful for detecting diabetic complications [142, 143].

A chemoattractant encoded by the RARRES2 gene, chemerin, also known as retinoic acid receptor responder 2, has recently been identified as a novel adipokine that regulates adipogenesis and homeostasis of adipocytes [88]. Increased excretion of urine albumin is intimately linked to glomerular dysfunction, a hallmark of the loss in renal function in diabetic kidney disease. Chemerin serum levels have been found to be negatively connected with estimated glomerular filtration rate and homocysteine level and positively connected with systolic blood pressure, elevated c‐reactive protein sensitivity, blood urea nitrogen, serum creatinine, insulin, and insulin resistance index. These findings suggest that circulating chemerin levels are independently correlated with renal function markers [16, 144].

The development of chronic kidney disease, especially in chronic inflammatory conditions, has been linked to elevated plasma levels of chemerin linked to renal dysfunction, abnormalities in glucose and lipid metabolism were noted [145]. According to research, 16%–25% of variations in serum chemerin levels are due to genetic factors, making them somewhat heritable [103]. Variants of the RARRES2 gene are linked to raised visceral fat mass and chemerin levels in nonobese people, as well as a higher risk of MetS [3, 146]. In addition, research has demonstrated a link between MetS and the minor polymorphism allele of rs17173608 chemerin [123, 147].

Researchers have shown that, relative to those without, plasma levels of chemerin are higher in diabetic patients with microangiopathic complications. And a higher level of plasma chemerin is an independent association between retinopathy and nephropathy in diabetic patients, not peripheral neuropathy [148].

### 5.3. Chemerin and MetS

Public health agencies, health clinicians, healthcare researchers, and the general public have been concerned by the dramatic rise in the incidence of childhood obesity [149]. Obesity in childhood is correlated with a variety of disorders [150, 151] and long‐term cardiovascular complications [152]. Furthermore, obese children appear to become obese adults [16, 153]. In order to prevent adult obesity and related metabolic disorders, research on obesity during childhood is particularly important for these reasons. Recent research has identified the important role that adipose tissue hormones, adipokines, play in complications associated with obesity. Of the adipokines, extensive studies have been carried out on leptin [14], adiponectin [154], and resistin [155], while another adipokine, chemerin, has been studied in recent years [156].

Chemerin expression was found to be increased in obese and T2D animals in adipose tissue, and chemerin has been reported to regulate adipogenesis [93]. In addition, recent studies have shown that serum chemerin is associated positively with body weight and HOMA‐IR in pediatric overweight patients [157] and effect factors of MetS in obese children [158]. In addition, other studies have also documented that chemerin may play a role in the development of cardiovascular diseases in children and adolescents [16, 159]. Collectively, these results demonstrate that chemerin may play a significant role in the regulation of obesity and MetS. In obese children and adolescents, however, there are few studies on chemerin, particularly in China.

The MetS encompasses a number of cardiometabolic risk factors and determinants, such as insulin resistance, glucose intolerance, central obesity, dyslipidemia, hypertension, and nonalcoholic fatty liver disease (NAFLD). Due to the ongoing obesity pandemic, MetS is becoming more and more commonplace globally. There will also be a threat to the prevalence of T2D and cardiovascular disease worldwide [3, 45]. An essential endocrine organ, adiposity regulates metabolism and energy homeostasis in addition to storing energy. Chemerin, an adipokine, was discovered lately, and its expression was elevated in obese individuals [93].

Regulation of specific immune cell migration, anti‐inflammatory effects on macrophages, and regulation of adipogenesis are some of the basic functions of chemerin. In a relatively small sample of human patients from Mauritius, circulating levels of chemerin have been found to be significantly associated with MetS traits [35, 91]. When Bozaoglu et al. tested the amount of chemerin in the blood, they discovered a strong correlation between chemerin plasma concentrations and blood pressure, plasma triglycerides, and BMI [15]. Treatment with metformin reduces serum chemerin levels in women with polycystic ovary syndrome [16, 160].

Physical inactivity is known as the risk factor of T2D [161], in obese people, aerobic activity has been shown to decrease adiposity and insulin resistance [3]. The MetS’s chemerin concentrations have not been shown to alter as a result of any prior lifestyle modifications, which could explain the association between insulin resistance and obesity. Changing one’s lifestyle to avoid being overweight, not exercising, and eating unhealthy food has been identified as a key component of managing MetS [162]. However, only a change of lifestyle will also not achieve clinically effective weight loss [16, 163].

MetS, a worldwide public health concern, makes people more susceptible to obesity, diabetes, and heart disease. Even though the underlying mechanisms are still unclear, adipokines have been crucial in gathering evidence. Chemerin is a recently identified adipokine that is encoded by the RARRES2 gene and is implicated in inflammation, adipogenesis, angiogenesis, and energy metabolism. Human BMI and biomarkers linked to obesity are substantially correlated with local and circulating levels of chemerin [16].

Animals with diabetes and obesity generally have higher levels of chemerin. Chemerin is linked to adipogenesis, glucose homeostasis, food intake, and body weight, according to previous research on function increase or loss. Adipokines are among the peripheral afferent signals that the brain’s hypothalamus integrates to control hunger and energy homeostasis. Chemerin increases food intake in seasonal animals by influencing tanycytes, which are hypothalamic stem cells. Chemerin causes angiogenesis, inflammation, and cell proliferation in peripheral adipose tissue, all of which contribute to obesity. There are contradictory studies about the relationship between chemerin and obesity and insulin resistance, despite the fact that chemerin signaling enhances pancreatic islet insulin output [3].

Given the relationship between chemerin and obesity comorbidities in humans, it is predicted that progress in translational research targeting chemerin would reduce metabolic disorders. The intriguing findings accumulated over the last ten years collectively demonstrate a crucial and complex function for chemerin in the regulation of energy balance, making it a viable option for the critically needed pharmaceutical treatment of obesity [3, 164].

Blood pressure, homeostasis, adipogenesis, glucose metabolism, and other biological processes have all been demonstrated to be impacted by adipokines since the discovery of leptin. This has led to the association of adipokines with MetS. Several studies have demonstrated that adipokine chemerin is essential for adipogenesis, which in turn affects adipose tissue control in relation to glucose homeostasis regulation and the emergence of obesity [16, 164].

### 5.4. Chemerin and NAFLD

NAFLD spans simple steatosis to steatohepatitis and cirrhosis and is closely linked to obesity, insulin resistance, and systemic inflammation. Several clinical studies report higher circulating chemerin in NAFLD and correlations with insulin resistance (HOMA‐IR), inflammatory markers, and disease severity [95–101]. Because chemerin is produced by both adipose tissue and liver, total circulating chemerin should be interpreted cautiously as a liver‐specific biomarker; isoform‐resolved and tissue‐specific studies are needed to clarify whether chemerin contributes to NAFLD pathogenesis or primarily reflects adipose/liver inflammation [15, 51].

### 5.5. Chemerin and Hypertension

Hypertension has been identified as an important component of MetS. Serum chemerin levels are often higher in patients with hypertension [165, 166]. Some studies report that this association persists after adjustment for metabolic risk factors, although residual confounding remains possible [165]. Chemerin may influence vasoconstriction and/or vascular smooth muscle cell behavior via CMKLR1 signaling, providing a plausible mechanistic link to elevated blood pressure [167–169].

Additionally, chemerin causes a dose‐dependent calcium influx in vascular smooth muscle cells, which underpins chemerin‐induced vasocontraction and hypertension, via activating the L‐type Ca^2+^ channel via Gi proteins, according to a recent study [170]. It was discovered that Y27632, a pyridine derivative, might target a Rho‐associated protein kinase (ROCK) and decrease ROCK‐mediated Ca^2+^ sensitization. The discovery that chemerin signals through the RhoA/ROCK pathway is supported by the ROCK inhibitor Y27632, which inhibits chemerin‐induced calcium influx and isometric contraction of smooth muscle cells [170, 171]. Thus, increased serum chemerin may cause vascular smooth muscle cells to vasoconstrict by activating ROCK and sensitizing Ca^2+^ [45].

It is possible that antihypertensive medications can directly affect how chemerin is processed, which complicates its involvement in hypertension. For example, the angiotensin‐converting enzyme (ACE) acts as a CP, breaking down chemerin 20–163 to inactive chemerin 152 [45]. Therefore, ACE inhibition would be expected to increase the concentration of more active isoforms of chemerin. Likewise, chymase (also a producer of angiotensin II) metabolizes chemerin into an active and inactive form [8, 172]. The ACE inhibitor fosinopril decreased serum and renal chemerin elevation in rat diabetic nephropathy triggered by streptozocin [169, 173]. Finally, the peroxisome proliferator–activated receptor gamma (PPARγ) agonists—rosiglitazone and pioglitazone [174], and the angiotensin receptor antagonist—irbesartan [175], minimize the increase in chemerin protein and chemerin 1 receptor observed in the kidney of the streptozocin‐induced diabetic rat. Whether chemerin levels were lowered as a result of the condition being treated or as a result of these medications altering chemerin processing is just unclear from these later research [45].

The classification of chemerin has evolved from chemokine to adipokine, indicating its involvement in a broad range of physiological processes. The cardiovascular system is one such system, particularly in connection with hypertension, where chemerin exerts a variety of impacts [45, 176].

Chemerin was bell‐shaped associated with diastolic blood pressure, while there was no correlation for systolic blood pressure after waist circumference adjustment. Interestingly, after excluding subjects taking antihypertensive agents, the correlation with systolic BP shifted to borderline meaning, implying that these medications affect the values of chemerin. A recent study therefore detected a decrease in chemerin levels after treatment with irbesartan in rats [175]. The nonlinear association with diastolic blood pressure changed to a positively linear one once participants on lipid‐lowering or antidiabetic medications were further excluded. Both classes of drugs are known to raise blood pressure in a protective way [169, 177] and have probably blunted a chemerin‐associated rise in BP in these subjects.

In addition, elevated levels of chemerin were correlated with higher risks of high blood pressure. Previous studies showed positive associations of chemerin with both blood pressure values [15] or systolic [178] as well as diastolic blood pressure [166] alone or for neither of the two [165]. The positive correlation of hypertension with chemerin was shown in two smaller studies [165, 179]. The relationship between chemerin and BP can be clarified by chronic inflammation associated with obesity since proinflammatory factors lead to the development of hypertension via endothelial and vascular smooth muscle cell dysfunction [180]. Furthermore, alterations in adipocyte size and quantity need the growth of pre‐existing capillary networks and the formation of new blood vessels through angiogenesis. CMKLR1 expression has been shown to be upregulated in human vascular endothelial cells in response to inflammatory cytokines [85, 169].

In addition, in these cells, chemerin successfully induced functional angiogenesis [85]. By showing that chemerin causes apoptosis in human endothelium, other studies, however, have shown the opposite effects on vascular smooth muscle cells [181] and murine cardiomyocytes [182]. The pathophysiology of cardiovascular disorders is thought to be significantly influenced by chemerin; however, further investigation into the intricate underlying mechanisms is required. Elevated chemerin levels may be linked to higher systolic blood pressure in obese children [183]. Chemerin is one of these adipokines of humans whose blood pressure has been positively correlated with plasma levels and is clinically known as a MetS marker [45, 176].

Mechanistically, chemerin signaling can influence vascular tone. In addition to the calcium‐dependent vasoconstrictor effects described above, chemerin has been reported to reduce NO‐mediated relaxation and cGMP signaling in vascular tissue [45, 103]. ChemR23 has been detected in human atherosclerotic lesions on smooth muscle and foam cells [104], supporting a potential contribution of chemerin signaling to vascular dysfunction in cardiometabolic disease.

#### 5.5.1. Gestational Hypertension and Preeclampsia

Pregnancy‐related hypertensive disorders have also been linked to chemerin. Several studies reported higher maternal serum chemerin in preeclampsia and persistence of elevated levels after delivery, with associations with postpartum hypertension [45, 124]. Prospective cohort data further suggest that third‐trimester chemerin may be associated with later hypertension after preeclampsia [169]. Because the BMI and metabolic risk factors can strongly confound these relationships, future studies should incorporate isoform‐resolved assays and carefully adjust for adiposity and renal function.

### 5.6. Cardiometabolic Disease

Chemerin is significantly expressed in WAT, and the expression is more significant in obese than in lean mice [86]. Chemerin is important in adipogenesis and angiogenesis [31, 184] and acts as a chemoattractant, attracting immune cells to sites of tissue damage. When synthetic chemerin–derived peptides were administered to mice, apoptotic cells and microbiological particles were phagocytosed by macrophages [185]. Higher levels of chemerin in the blood have been connected to human cardiovascular events, dyslipidemia, and inflammatory indicators [140]. There are three known genetic loci linked to chemerin concentrations, which are located close to RARRES2, which codes for vaspin, and three more genes [184, 186]. However, in the largest genomewide association studies to date, these loci are not associated with cholesterol levels, coronary artery disease risk, or other cardiometabolic features. Consequently, the available genetic evidence does not support a causal relationship between chemerin and cardiometabolic risk [187–189].

T2D, cardiovascular disease, and mortality were all linked to MetS, a condition marked by a number of cardiometabolic risk factors [94]. MetS pathophysiology is complicated and incompletely elucidated. The distribution of ectopic fat, insulin resistance, and inflammation, however, are all main pathological players in the MetS components [190]. The main source of adipokines in a range of organs and tissues is adipose tissue signals to targets, which alter blood pressure, immunological function, glucose and lipid metabolism, and energetic balance [191]. In adipose tissue dysfunction, like obesity and MetS, adipokine secretion is altered, which can lead to obesity‐associated diseases [192, 193]. Although leptin and adiponectin are known to have negative and positive effects on cardiometabolic health, respectively [194, 195], the function of other adipokines such as omentin and chemerin as cardiometabolic risk markers remains uncertain [89, 164, 189].

**Figure 3:**
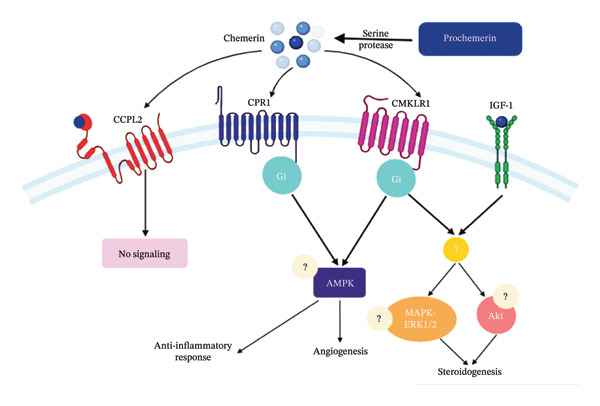
Schematic summary of selected chemerin‐activated signaling pathways implicated in tumor‐cell migration, proliferation, and invasion (adapted from Refs. [78, 225–227]).

As of right now, chemerin’s function in cardiometabolic health is unclear. The adipokine was linked to both anti‐inflammatory and proinflammatory properties [164, 196]. Monocyte adherence to TNF‐α–stimulated endothelial cells and vascular cellular adhesion molecule‐1 (VCAM‐1) expression are inhibited by TNF‐α [197], and decreasing TNF‐α, IL1‐β, IL‐6, and C‐X‐C motif chemokine ligand 11 (CXCL11) production, chemerin can exert anti‐inflammatory effects [189]. Other research suggests that chemerin has a proinflammatory effect by boosting the expression of cell adhesion molecules, improving monocyte/macrophage adhesion to endothelial cells, and influencing the expression of the pathways NF‐κB, MAPK, and phosphatidylinositol‐3‐kinase/protein kinase B (PI3K/Akt). It also promotes the migration of NL cells, dendritic cells, and monocytes/macrophages [198] (Figure 3). Whether chemerin causes insulin resistance to rise or fall is likewise unknown. Additionally, it is unclear whether chemerin causes insulin resistance to rise or fall. In 3T3‐L1 adipocytes, chemerin has been shown to regulate insulin‐stimulated glucose uptake [199, 200], yet another study stated the contrary [201, 202]. Chemerin may also affect the role of β‐cells [203]. Chemerin injection caused obese/diabetic mice to become more glucose intolerant, have lower serum insulin levels, and absorb less glucose from their tissues [30]. Positive associations between chemerin and adiposity and low‐grade inflammation were seen in recent studies, supporting the idea that it may be harmful to cardiometabolic health [189].

Numerous indicators, such as those related to insulin resistance, inflammation, metabolism, and obesity, were used to investigate the connection between adipokines and cardiometabolic health. Circulating adipokine levels may be measured to evaluate cardiometabolic risk. More research is required to fully comprehend the pathophysiology of T2D, cardiovascular disease, and MetS and how adipokines can be used to forecast the occurrence of these morbidities in the future [45, 94].

## 6. Association of Chemerin With Other Nonmetabolic Diseases

### 6.1. Chemerin and RA

Synovitis and joint erosion are hallmarks of RA, a systemic inflammatory disease that also manifests extra‐articular symptoms as pulmonary and vascular dysfunction. Approximately 0.5%–1% of people are impacted [204, 205]. With a female‐to‐male ratio between 2:1 and 4:1, it is a sneaky condition that typically manifests as symmetrical joint swelling in the fourth and fifth decades, peaking in incidence [206]. Although several studies link environmental and genetic factors to the disease’s etiology, the exact origin of RA is still unknown [207]. In addition to stiffness, this chronic autoimmune disease is characterized by heated, painful joints that swell, especially in the morning when you get up or after a lengthy period of inactivity [208, 209].

RA is the most prevalent autoimmune disease, and patients with RA exhibit immune cell infiltrates in their synovial tissues [210, 211]. A key factor in the development and maintenance of joint inflammation is the production of certain inflammatory markers by different cells, including dendritic cells, fibroblast‐like synoviocytes, and monocytes/macrophages [212, 213]. Improved diagnostic biomarkers are constantly needed for RA early identification. Research has focused on these trace elements [214] and various proteins [23].

As a pleiotropic organ with a focus on endocrine functions, WAT can include a variety of hormones and other proteins that are implicated in immunological and inflammatory responses, as well as physiological and pathological processes [215]. One risk factor for autoimmune conditions like RA is obesity [214] Since adipose tissue releases adipokines capable of creating a low‐grade inflammatory environment [216, 217]. Recently, chemerin has become a prominent adipokine involved in the immunological response. Subjects who are overweight or obese have higher amounts of chemerin than those who are normal weight, and their plasma values drop following dieting [218]. Through its interaction to the CMKLR1 receptor, chemerin exhibited both pro‐ and anti‐inflammatory properties [23, 35]. Chemerin is also engaged in innate and adaptive immunity as a chemoattractant for macrophages, NL cells, and specific subsets of dendritic cells [23], in addition to being an adipokine [202]. One of the potential future inflammatory response biomarkers is chemerin [23]. It is a protein of 16 kDa isolated from ascetic fluids of patients with ovarian cancer and synovial exudate of patients with RA.

Adipogenesis, angiogenesis, and inflammation were controlled by chemerin, which grew as RA lasted longer. Serum levels of this adipokine are also linked to elements of the MetS, such as high blood pressure, plasma TG, and BMI [219]. Chemerin serum levels are higher in RA patients and are linked to more systemic inflammation than fat, according to another study [220]. Such findings indicate chemerin as an important biomarker of the activity of the disease [220]. The inflammatory biomarkers of visfatin and chemerin levels can be utilized to diagnose RA patients. When controlling for other cofounders, chemerin and visfatin in particular are frequently linked to RA disease [16, 221].

Pigment epithelium–derived factor (PEDF) and chemerin may be indicators of obesity and inflammation in people with RA. Chemerin’s dual role of mimicking inflammation and metabolism, as well as the association between chronic inflammation and obesity, suggests that it is linked to the activity and responsiveness to therapy of RA disease. Accordingly, a minimum 5% decrease in the BMI, as shown by chemerin regulation, allows for improved disease management without altering RA medication [16, 222].

### 6.2. Chemerin and Psoriasis and Other Inflammatory Skin Disorders

Chemerin is expressed in normal skin and contributes to cutaneous homeostasis, including antimicrobial defense [87]. In psoriasis, chemerin biology appears context‐dependent: Early work suggested reduced chemerin in psoriatic epidermis compared with normal skin, whereas subsequent studies reported increased chemerin expression in lesions and/or higher circulating chemerin in patients [1, 223, 224]. Mechanistically, chemerin can recruit plasmacytoid dendritic cells and other innate immune populations via CMKLR1, potentially contributing to inflammatory amplification in susceptible skin [9, 30, 83].

Clinical studies have linked higher chemerin to psoriasis severity and to cardiometabolic comorbidities, although results are heterogeneous and confounded by obesity, systemic inflammation, and treatment exposure. Standardized chemerin assays (including isoform‐resolved measurements) and well‐controlled cohort studies are needed to clarify whether chemerin is a driver of cutaneous inflammation or primarily reflects systemic metabolic‐inflammatory status.

### 6.3. Chemerin and Cancer

Compared to healthy controls, Xu et al. found that nonsmall cell lung cancer patients had significantly higher serum chemerin levels [228]. Furthermore, Lu et al. [229] confirmed similar findings regarding the association between serum chemerin and oral premalignant lesions and oral squamous cell carcinoma [229]. Sotiropoulos et al., however, found no discernible difference between prostate cancer patients and healthy controls in terms of circulating levels of chemerin [230]. Additionally, there were conflicting results about the association between chemerin levels and cancer risk. Furthermore, the majority of research merely explains the link between circulating chemerin and a certain type of cancer. To the best of our knowledge, numerous reviews have established links between circulating chemerin and the risk of developing different types of cancer [230, 232]. Nevertheless, no meta‐analysis has been done. Research is still needed to determine whether circulating chemerin has fundamental impacts on cancer or whether it is a useful biomarker for cancer diagnosis [233, 234].

The biochemical mechanism linking circulating levels of chemerin to an elevated risk of cancer could be explained as follows. According to reports, chemerin aids angiogenesis by collaborating with CMKLR1 [235, 236]. In recent years, there has been a widespread belief that chemerin promotes the chemoattraction of different immunocytes whose receptors are expressed on malignant tumor cells in the tumor microenvironment [237, 238]. Using its antibacterial qualities, Banas et al. found that chemerin might be utilized to control intestinal microbial activity, which would impact patients with colorectal cancer [239]. Additionally, chemerin may influence the development of gastric cancer by producing more VEGF, MMP‐4, and IL‐6 or by boosting the activity of the MAPK (MEK‐ERK, MKK3/6‐p38) pathway [233]. Additional research has shown that chemerin‐mediated effects increased MMP activity, which in turn exacerbated the aggressiveness of associated malignancies [188, 234]. One or more of the aforementioned processes could explain chemerin’s impacts on tumor growth.

However, chemerin has been shown to inhibit hepatocellular carcinoma cell motility, invasion, and metastasis by interfering with the phosphatase and tensin homolog (PTEN)‐CMKLR1 connection and then upregulating PTEN expression and phosphatase activity [240]. Additionally, chemerin inhibits the growth of myeloid‐derived suppressor cells (MDSCs) and rebuilds antitumor IFN‐γ + by negatively regulating granulocyte‐macrophage colony‐stimulating factor (GMCSF) and IL‐6. This prevents tumor angiogenesis [241, 242]. In these situations, chemerin is secreted by tumor cells, combining its several roles, but the host system will compensate to produce an immune response that fights the tumor [125]. In cancer patients, this could explain the increased level of circulating chemerin, even if the role of chemerin was protective [233, 234].

According to recent research, circulating chemerin levels in cancer patients were noticeably greater than in the control group. This suggests that there is a strong correlation between high levels of circulating chemerin and the risk of developing cancer. However, the majority of the fundamental mechanisms underlying how the chemerin level affects cancer risk were only conjecture since they lacked a solid foundation. Therefore, more straightforward and convincing mechanisms ought to be covered in future studies. Moreover, each cancer type’s specificity must be taken into account for increased clinical significance [233].

**Figure 4:**
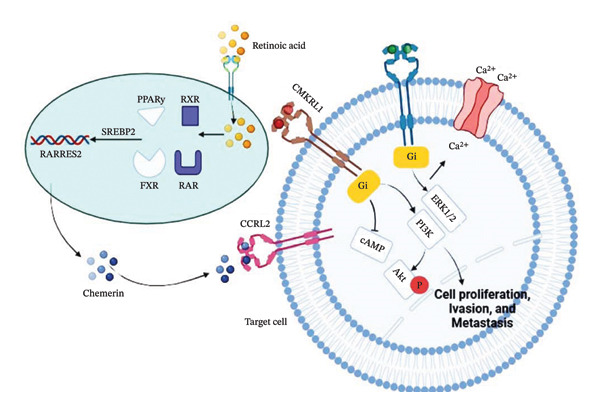
Effects of chemerin to induce cell proliferation, invasions, and metastasis and effects of retinoic acid to enhance the expression of chemerin.

Chemerin possesses anti‐inflammatory and proinflammatory properties, depending on the model being examined [84] (Figure 4). It also contributes to the development of cancer, and once more, both pro‐ and antitumorigenic effects have been documented [84, 231]. Chemerin controls the movement of immune cells and is chemotactic for a variety of cells. Chemerin‐induced recruitment of natural killer cells inhibited melanoma [237]. In HCC experimental models, the physiologically highly active murine chemerin isoform chemerin‐156 prevented tumor growth and metastasis [240, 243]. Chemerin’s tumor‐inhibitory impact in HCC was diminished in Rag1‐/‐animals, indicating a role for T cells [234, 244].

## 7. Conclusion

Chemerin is a multifunctional adipokine/chemoattractant produced mainly by WAT, liver, and placenta and activated by proteolytic processing into isoforms with distinct bioactivity. Via CMKLR1/ChemR23, GPR1, and CCRL2, chemerin contributes to physiological regulation of immune cell trafficking, adipose tissue biology, glucose and lipid homeostasis, and vascular function.

Accumulating experimental and clinical evidence links altered chemerin signaling to major metabolic disorders (obesity, insulin resistance, T2D, MetS, NAFLD, and hypertension) and selected nonmetabolic conditions (inflammatory disorders and cancer), but the results are heterogeneous and often confounded by adiposity, inflammation, and assay variability. Future research should prioritize standardized measurement of total and isoform‐specific chemerin, clarify tissue sources and receptor‐specific mechanisms, and test clinical utility in well‐designed prospective cohorts and interventional studies.

**Nomenclature**
ACE: angiotensin‐converting enzyme
AgRP: agouti‐related peptide
ARC: arcuate nucleus
BMI: body mass index
CCL: CC‐chemokine ligand
CCRL2: CC‐chemokine receptor‐like 2
cGMP: cyclic guanosine monophosphate
CMKLR1: chemokine‐like receptor 1 (ChemR23)
ChemR2: chemerin receptor 2
ChemR3: chemerin receptor 3
CPB: carboxypeptidase B
CPN: carboxypeptidase N
CSF: cerebrospinal fluid
CRP: C‐reactive protein
CXCL11: C‐X‐C motif chemokine ligand 11
DCs: dendritic cells
DPP4: dipeptidyl peptidase 4
EMSA: electrophoretic mobility shift assay
ERK1/2: extracellular signal‐regulated kinases 1/2
FFA: free fatty acids
GDM: gestational diabetes mellitus
GPR1: G protein–coupled receptor 1
HbA1c: glycated hemoglobin
HDL: high‐density lipoprotein
HOMA‐IR: homeostasis model assessment of insulin resistance
ICAM‐1: intercellular adhesion molecule 1
IL: interleukin
IL‐1β: interleukin‐1beta
IL‐8: interleukin‐8
MAPK: mitogen‐activated protein kinase
MetS: metabolic syndrome
MMPs: matrix metalloproteinases
MMP: matrix metalloproteinase
NAFLD: nonalcoholic fatty liver disease
NK cells: natural killer cells
NO: nitric oxide
NF‐κB: Nuclear factor kappa‐light‐chain‐enhancer of activated B cells
p38 MAPK: p38 mitogen‐activated protein kinase
PPARγ: peroxisome proliferator–activated receptor gamma
PEDF: pigment epithelium–derived factor
PTEN: phosphatase and tensin homolog
PI3K/Akt: phosphatidylinositol‐3‐kinase/protein kinase B
POMC: pro‐opiomelanocortin
PVS: portal venous system
RA: rheumatoid arthritis
RARRES2: retinoic acid receptor responder 2
ROCK: Rho‐associated coiled‐coil kinase
SCCOT: squamous cell carcinoma of the tongue
SFs: synovial fibroblasts
SREBP2: sterol regulatory element–binding protein 2
T1D: Type 1 diabetes mellitus
T2D: Type 2 diabetes mellitus
TIG2: tazarotene‐induced gene 2
TLRs: Toll‐like receptors
TNF: tumor necrosis factor
VEGF: vascular endothelial growth factor
VCAM‐1: vascular cellular adhesion molecule‐1
WAT: white adipose tissue

## Author Contributions

Noha A. Ahmed: writing–original draft, writing–review and editing, and visualization. Aida A. Hussein: writing–review and editing and visualization. Rehab G. Khalil: writing–original draft, writing–review and editing, and visualization. Nour Y. S. Yassin: writing–original draft, writing–review and editing, and visualization. Mohamed A. Abdelaziz: writing–review and editing and visualization. Ayman I. Geddawy: writing–review and editing and visualization. Osama M. Ahmed: conceptualization, writing–original draft, writing–review and editing, and visualization.

## Funding

This research received support from Prince Sattam bin Abdulaziz University under Project No.: PSAU/2023/R/1444.

## Ethics Statement

The authors have nothing to report.

## Conflicts of Interest

The authors declare no conflicts of interest.

## Data Availability

The authors have nothing to report.
